# Sedentary Behaviours in Mid-Adulthood and Subsequent Body Mass Index

**DOI:** 10.1371/journal.pone.0065791

**Published:** 2013-06-07

**Authors:** Snehal M. Pinto Pereira, Chris Power

**Affiliations:** Centre for Paediatric Epidemiology and Biostatistics, UCL Institute of Child Health, University College London, London, United Kingdom; Charité University Medicine Berlin, Germany

## Abstract

**Objectives:**

It is unclear whether sedentary behaviour, and the domain in which it occurs, is related to body mass index (BMI) change. We aim to elucidate whether sedentary behaviour is prospectively related to BMI change using markers from three domains (leisure, work and commuting).

**Methods:**

Among employed 1958 British birth cohort members (n = 6,562), we analysed whether TV-viewing, work sitting (six categories: 0 h/d to >4 h/d) and motorised commuting (at 45 y) were related to BMI (at 45 y and 50 y) and BMI change 45–50 y, after adjusting for lifestyle and socioeconomic factors.

**Results:**

Per category higher TV-viewing, 45 y and 50 y BMI were higher by 0.69 kg/m^2^ (95% CI: 0.59,0.80) and 0.75 kg/m^2^ (0.64,0.86) respectively. A category higher TV-viewing was associated with 0.11 kg/m^2^ (0.06,0.17) increased BMI 45–50 y, attenuating to 0.06 kg/m^2^ (0.01,0.12) after adjustment. There was no trend for work sitting with 45 y or 50 y BMI, nor, after adjustment, for BMI change. However, those sitting 2–3 h/d had greater BMI gain by 0.33 kg/m^2^ (0.10,0.56) compared to those sitting 0–1 h/d. Associations between TV-viewing and BMI change were independent of work sitting. Motorised commuting was associated with 45 y, but not 50 y BMI or change.

**Conclusions:**

TV-viewing is associated with BMI gain in mid-adulthood; evidence is weaker for other sedentary behaviours.

## Introduction

There is increasing evidence that sedentary behaviour contributes to multiple health outcomes, including all-cause mortality, separately from physical activity [1]. It is suspected that sedentary behaviour and physical activity may be different constructs affecting health via independent pathways, and not necessarily as opposing ends of the same spectrum. Given the mounting evidence, physical activity guidelines now include recommendations to minimise the amount of time spent being sedentary [2], [3]. Yet, sedentary behaviours are ubiquitous and on the rise: in the USA, adults spend 55% of their waking hours being sedentary [4], and time spent watching TV increases year on year [5], [6]. Hence sedentary behaviour has emerged as a key modifiable behaviour for which there is a need to elucidate the pathways through which it leads to adverse health.

Sedentary behaviour and physical activity may independently contribute to adult obesity, which has increased almost universally [7]. For example, in the USA, prevalence of obesity increased from 26.9% to 32.0% in men and from 33.2% to 35.2% in women between 1999 and 2008 [8], while, in England, obesity prevalence increased from 13% to 26% in men and from 16% to 26% in women between 1993 and 2010 [9]. In addition to compromising the populations' healthy, productive life span, obesity places a significant financial burden on health systems. If trends continue, by 2030, increases in obesity related diseases are projected to add to health-care costs by $48–66 billion a year in the USA and by £1.9–2 billion a year in the UK [10]. Hence, preventing excess body mass index (BMI) gain is an important public health and economic goal. To that end it is necessary to identify modifiable behaviours that contribute to BMI gain. Prospective studies on sedentary behaviour and subsequent adiposity have considered either non-occupational sitting (consisting mainly of TV-viewing [11]–[17]) or an overall measure of sitting (either from device-based measures [18] or questionnaires [19]). The domain within which sedentary behaviour occurs is of potential importance [20] and a focus exclusively on TV-viewing or overall sitting time, may obscure associations between sedentary behaviour in specific domains and subsequent adiposity change. TV-viewing may be the most common sedentary behaviour that adults engage in; but it occupies a small proportion of the waking day. Most adults are sedentary for 7–10 h/d, with work sitting often occupying much of this time [1]. Furthermore, sedentary behaviours have different patterns of association with socio-demographic and lifestyle factors linked with adverse health. In a cross-sectional study of adults we have previously shown a trend of adverse socio-demographic and lifestyle characteristics with increasing levels of TV-viewing, but a trend in the opposite direction for work sitting. For example, we observed a trend of low fruit and high chips consumption with increasing levels of TV-viewing, whereas opposing trends were seen for work sitting. Such observations suggest that sedentary behaviours from different domains are confounded in different ways [20].

In the present study, using a nationwide cohort we aim to establish whether sedentary behaviour is associated with subsequent BMI change in mid-adulthood, ages 45 y to 50 y, and whether associations vary by the indicator of sedentary behaviour, namely TV-viewing, work sitting and method of travel to work.

## Materials and Methods

Ethical approval was given by the South East Multi-Centre Research Ethics Committee and written informed consent was obtained from cohort members. The 1958 British Birth Cohort consists of over 17,000 participants followed-up since birth during one week in March 1958 across Britain [21]. Participants in mid-adulthood are broadly representative of the total surviving cohort [22]. At 44–45 y, 9,377 participated in a biomedical survey of whom 7,660 were in paid employment. At the most recent contact (50 y), 9,790 individuals participated, of whom 6,799 were in paid employment at 45 y. In this study, the target sample available for analysis was 6,799 comprising those who participated at 50 y and who were in paid employment at 45 y, restricted to 6,562 participants with a BMI measurement at 50 y.

### BMI measures

At 45 y height and weight were measured using Leicester portable stadiometers and Tanita solar scales respectively. Self-reported weight was used if accurate measurements or consent for measurement were unavailable (n = 40). If 45 y measured height was unavailable either 33 y measured height (n = 32) or self-reported height (n = 3) was used. At 50 y weight was self-reported. BMI was calculated as weight/height^2^ (kg/m^2^) at 45 y and 50 y, using 45 y height for both ages.

### Explanatory variables

TV-viewing and work sitting at 45 y were assessed using the previously validated EPIC-Norfolk physical activity questionnaire (EPAQ-2) [23], with minor modifications [24]. Participants reported average TV-viewing, outside of work, in six categories from none (0 h/d) to >4 h/d. Work sitting was derived as a continuous measure from the question “how many h/wk do you sit doing light work” and then categorised into six levels, similar to TV-viewing from 0 h/d to >4 h/d. At 45 y, participants reported how they usually travelled to work (i.e. using motorised transport, bicycle or walking); a binary variable of always by motorised transport vs. other was constructed.

### Potential confounding and mediating factors

Potential confounding and mediating factors from across the life-course were identified from previous studies of associations between sedentary behaviours and adiposity [12], [19], [25] and specifically in this population [26], [27]. Birthweight, measured in ounces and pounds, was converted into kilograms. Indicators of lifetime socioeconomic position included father's occupational class at birth (or at 7 y if missing) which was categorized into 4 groups using a standard method of categorising occupations in the UK, the Registrar General's Social Classification: I (professional) or II; IIINM; IIIM; and IV, V (unskilled), or single mother. Participant's occupational class at 42 y (or at 33 y if missing) was categorized similarly. Educational level was recorded prospectively to 42 y and categorized into five groups from no qualifications to degree or higher. Physical activity during leisure, was ascertained by self-report of frequency of participation in sports or other regular activity at 42 y and grouped into four categories from ≤2–3 times/mo to 4–7times/wk. Smoking was self-reported at 42 y (or 33 y if missing) and categorized as never, ex-smoker, or current smoker. Pre-existing health conditions were identified as longstanding illness, disability or infirmity limiting daily activities at 42 y. Participants reported food consumption frequency at 42 y, including chips (three groups, <1 d/wk to 3+d/wk), sweets/chocolates, biscuits/cake and fruit (four groups,<1 d/wk to >1+times/d). Alcohol use at 45 y, using the Alcohol Use Disorders Identification Test questionnaire [28], was categorized into four groups from non- or light-drinker to very heavy drinker (>21 drinks/wk).

### Statistical analysis

We examined associations for TV-viewing and work sitting separately with 45 y and 50 y BMI using linear regression models and then assessed whether prospective associations with BMI change 45–50 y were explained by life-style and socioeconomic factors from across the life-course. In preliminary analysis, we tested whether associations for TV-viewing or work sitting with BMI change differed by gender using an interaction term between gender and the sedentary behaviour. No significant interactions were found hence models are presented for both genders combined. Preliminary analyses also included tests of deviation from linearity, using the likelihood ratio test, and where there was no evidence of non-linearity we report linear trends. Analyses were undertaken with three levels of adjustment: first, for BMI at 45 y and 50 y, adjusted for gender only (Model 1), second, to investigate BMI change, we adjusted associations with 50 y BMI for BMI at 45 y (Model 2), third, to determine whether the associations with BMI change were explained by life-style and socioeconomic characteristics we additionally adjusted for the factors described above (Model 3). To assess whether associations of TV-viewing and work sitting with BMI change were independent, models were repeated with both sedentary behaviours simultaneously. Next, we examined associations between commute to work and BMI using the above described analysis strategy. All analyses were repeated using weight rather than BMI as the outcome variable, with adjustment for height. Results were similar to those seen for BMI, hence only the later are presented.

Most (79%) cohort members had no missing data, while 13% had missing data on ≥5 variables, educational achievement was not missing for any cohort member. To minimize data loss, missing covariates were imputed using multiple imputation chained equations, using current guidelines [29]. Imputation models included all model variables plus previously identified key predictors of missingness: cognitive ability and behaviour at 7 y, social class at birth and in adulthood [22]. Regression analyses were run across 10 imputed datasets. Imputed results were similar to those using observed values; the former are presented here. Analyses were conducted using STATA (Version 11, Stata Corp) and two-sided testing was performed using a significance level of 0.05.

## Results

At 45 y, the distribution of time spent watching TV and sitting at work differed (Table 1). Approximately 14% of the population watched TV for ≤1 h/d while, 35% sat at work for ≤1 h/d, most of whom (21%) sat at work for 0 h/d; while only 7% of the population watched TV for >4 h/d, 35% sat at work for >4 h/d. Most participants (77%) always used motorised transport to commute to work. Mean increase in BMI between 45 y and 50 y was 0.25(95% CI: 0.17, 0.34)kg/m^2^ in men (p<0.001), there was no difference in women.

**Table 1:** Mean (SD) Body mass index (BMI) at 45 y and 50 y and 45 y Sedentary Behaviour (N, %) for Participants in the Study (N = 6,562)*.

	Total		Men		Women	
	N	Mean(SD)	N	Mean(SD)	N	Mean(SD)
BMI(kg/m^2^) at 45 y	6540	27.28(4.84)	3310	27.86(4.30)	3230	26.69(5.27)
BMI(kg/m^2^) at 50 y	6562	27.41(5.08)	3319	28.12(4.68)	3243	26.69(5.38)
TV-viewing(h/d)(45 y)		%		%		%
0	24	0.37	12	0.36	12	0.37
0–1	888	13.67	409	12.44	479	14.94
1–2	2372	36.53	1,209	36.77	1,163	36.28
2–3	1918	29.53	971	29.53	947	29.54
3–4	821	12.64	444	13.50	377	11.76
>4	471	7.25	243	7.39	228	7.11
Sitting at work(h/d)(45 y)						
0	1,347	20.78	586	17.93	761	23.69
0–1	907	13.99	364	11.13	543	16.91
1–2	664	10.25	320	9.79	344	10.71
2–3	859	13.25	394	12.05	465	14.48
3–4	439	6.77	189	5.78	250	7.78
>4	2,265	34.95	1,416	43.32	849	26.43
Commute to work (45 y)						
Always by motorised transport	4,968	76.88	2,632	80.39	2,336	73.27
Other	1,494	23.12	642	19.61	852	26.73

SD: Standard deviation.

* N varies due to missing data.

There was a linear trend of higher 45 y and 50 y BMI with higher TV-viewing at 45 y: for each category increase in TV-viewing, 45 y BMI was higher by 0.69(0.59, 0.80)kg/m^2^ and 50 y BMI was higher by 0.75(0.64, 0.86)kg/m^2^ (adjusting for gender, Model 1, Table 2). Gain in BMI 45–50 y was greater by 0.11(0.06, 0.17)kg/m^2^ per category higher TV-viewing (Model 2, Table 2). Associations remained, although attenuated, after adjusting for life-style and socioeconomic factors (Model 3, Table 2).

**Table 2:** Mean (95% CI) 45 y and 50 y Body mass index (BMI) and BMI change by TV-viewing, Work Sitting (h/d) and motorised commuting at 45 y (N = 6,562)*.

45 y Sedentary behaviour	45 y	50 y		45–50 y change
TV-viewing (h/d)	Model 1^a^	Model 1^a^	Model 2^b^	Model 3^c^
0	−0.25 (−2.18,1.67)	0.11 (−1.89,2.12)	0.34 (−0.64,1.32)	0.32 (−0.66,1.30)
0–1	Referent	Referent	Referent	Referent
1–2	0.90 (0.53,1.27)	0.91 (0.53,1.30)	0.09 (−0.10,0.28)	0.05 (−0.14,0.24)
2–3	1.61 (1.23,1.99)	1.82 (1.42,2.21)	0.35 (0.16,0.55)	0.26 (0.06,0.46)
3–4	2.19 (1.73,2.64)	2.19 (1.72,2.66)	0.20 (−0.03,0.43)	0.04 (−0.19,0.28)
>4	2.83 (2.29,3.36)	3.09 (2.53,3.65)	0.52 (0.24,0.79)	0.32 (0.03,0.60)
Per unit increase	0.69 (0.59,0.80)	0.75 (0.64,0.86)	0.11 (0.06,0.17)	0.06 (0.01,0.12)

Sitting at work (h/d)				
0	0.77 (0.36,1.18)	0.88 (0.45,1.31)	0.17 (−0.03,0.38)	0.07 (−0.14,0.28)
0–1	Referent	Referent	Referent	Referent
1–2	−0.10 (−0.58,0.39)	−0.13 (−0.64,0.37)	−0.04 (−0.29,0.20)	−0.01 (−0.26,0.23)
2–3	0.11 (−0.34,0.56)	0.40 (−0.07,0.87)	0.30 (0.08,0.53)	0.33 (0.10,0.56)
3–4	0.24 (−0.31,0.79)	0.16 (−0.41,0.73)	−0.06 (−0.33,0.22)	−0.03 (−0.31,0.25)
>4	0.27 (−0.10,0.65)	0.16 (−0.24,0.55)	−0.09 (−0.28,0.10)	−0.02 (−0.21,0.18)
Per unit increase	N/A	N/A	−0.04 (−0.07,−0.01)	−0.01 (−0.04,0.02)

Commute to work				
Motorised transport	0.33(0.05,0.61)	0.25(−0.04,0.55)	−0.05 (−0.19,0.09)	−0.05(−0.19,0.10)
Other	Referent	Referent	Referent	Referent

CI: confidence interval.

* Results based on imputed data.

a Model 1 adjusted for gender.

b Model 2 adjusted for gender and 45 y BMI.

c Model 3 adjusted for factors in Model 2 plus birthweight, social class at birth and in adulthood, education level, leisure time physical activity, smoking, longstanding illness limiting daily activity, dietary factors (chips, sweets, cake, fruit) and alcohol consumption.

There was no linear trend between time spent sitting at work and 45 y and 50 y BMI (Model 1, Table 2), but a negative trend with BMI change 45–50 y, i.e. BMI loss was 0.04(0.01, 0.07)kg/m^2^ per category higher sitting at work. This association was eliminated after adjustment for life-style and socioeconomic factors (Model 3, Table 2). However, those sitting at work for 2–3 h/d had a greater BMI gain by 0.33(0.10, 0.56)kg/m^2^ compared to those sitting at work for 0–1 h/d. When both TV-viewing and work sitting were modelled simultaneously, associations of each sedentary behaviour with BMI change remained largely unaltered (data not shown).

Those always commuting by motorised transport at 45 y had an average 0.33(0.05, 0.61)kg/m^2^ higher 45 y BMI than those who commuted by other means (adjusting for gender, Model 1, Table 2). However, there was no prospective association between commuting and 50 y BMI or BMI gain 45–50 y.

## Discussion

We found, firstly, a dose-response relationship between TV-viewing at 45 y and BMI at the same age and five years later at 50 y, while, there was no evidence of such dose-response relationships for work sitting. Secondly, TV-viewing was associated with BMI gain 45–50 y e.g. with a ∼0.11 kg/m^2^ gain per category higher TV-viewing, with attenuation to ∼0.06 kg/m^2^ in adjusted models, suggesting that there is confounding and mediation by factors such as social class, education, leisure time physical activity and diet. However, associations between TV-viewing and BMI gain were independent of time spent sitting at work. There was no trend in BMI gain with work sitting after adjustment for covariates, but the greater BMI gain among those sitting at work for 2–3 h/d by 0.33 kg/m^2^ compared to 0–1 h/d is consistent with a detrimental effect of being sedentary. Thirdly, we found no relationship between method of commute to work at 45 y and subsequent BMI.

### Methodological considerations

Study strengths include, first, the availability of information on three major domains of sedentary behaviour (leisure, work and commuting), whereas previous studies have focused mainly on TV-viewing [11]–[13], [25]. Second, our prospective design is better suited than cross-sectional studies, to assess the temporal order of association between sedentary behaviour and BMI. Third, we were able to adjust for several life-style and socioeconomic characteristics, some pertaining to earlier life-stages. Fourth, the nationwide coverage of the cohort increases the applicability of the findings to the general population, although restricted to those in paid employment, as imposed by a study involving both leisure and work-based sedentary indicators. Nonetheless, study limitations are acknowledged. Sedentary behaviour is self-reported rather than measured objectively. However, measures such as questionnaires can be informative in identifying the domains in which sedentary behaviour occurs which is not available from objectively measured data. Validation of the questionnaire, reported previously, [23] was for the EPAQ-2 questionnaire as a whole in relation to daytime energy expenditure, rather than for specific sedentary behaviours. Questions on time spent watching TV and work sitting had different formats: for TV-viewing participants responded using pre-defined categories (h/d), whereas they estimated total h/wk sitting doing light work. Time spent watching TV maybe recalled with less error and bias (due to set times for specific programmes) than time spent sitting at work. Repeatability of the EPAQ-2 questionnaire for work activity (of which work sitting is a sub-component) was found to be weaker than that for TV-viewing [23]. TV-viewing and work sitting may therefore not be measured with the same precision, which may in part explain the lack of trend between work sitting and BMI, while a trend was observed for TV-viewing. No data were available on time spent in sedentary travel to work to assess potential dose-response relationships. Moreover, there may be differences in time spent in each behaviour between weekdays and weekends which we were unable to capture. The development of more specialised sedentary measures is therefore desirable, that would allow for distinctions between separate domains [30]. Weight was measured at 45 y but self-reported at 50 y and the degree of measurement error or bias may vary. Self-reports tend to underestimate actual weight [31] implying that weight gain may be underestimated in our study. Such underestimation, at least for women, is suggested by comparison of our study showing no increase in average BMI 45 y–50 y and measurements obtained in the Health Survey for England (HSE), indicating a 0.48 kg/m^2^ gain 45 to 50 y between 2003 and 2008; whereas for men we found mean gain in 45–50 y BMI to be 0.25 kg/m^2^, the corresponding HSE value was 0.03 kg/m^2^. However, our study was restricted to those in paid employment at 45 y, and other differences in study design could account for discrepancies in BMI gain: HSE had a smaller sample of 45 y and 50 y-olds, did not include Wales or Scotland and did not measure the same individuals. BMI is highly influenced by height, hence we used measured height for calculation of 45 y and 50 y BMI. We acknowledge also, that despite adjustment for multiple factors, it is possible that there is unmeasured or residual confounding (for example, by physical activity from domains outside of leisure or additional dietary factors). Finally, sample attrition occurred over the period of follow-up, although previous work has shown mid-adulthood respondents to be broadly representative of the surviving cohort [22] and further loss due to missing information was handled by multiple imputation.

### Interpretation and comparison to other studies

Our work adds to the limited prospective evidence, as highlighted in a recent review [1], on associations between sedentary behaviour and BMI gain in adults, using multiple sedentary behaviour indicators. Our findings of a positive trend between TV-viewing at 45 y and subsequent BMI and, less consistently, for work sitting, are specific to mid-adulthood. Although we cannot extrapolate to BMI gains at other ages, there are advantages to following a single age group over time because BMI varies with age, increasing until late adulthood, and then decreasing [9]. Most previous prospective studies include populations with wide age ranges [11]–[13], [17], [19], [25] which may not be the most appropriate design for investigating associations of sedentary behaviour with adiposity gain. Some studies on sedentary behaviour and subsequent overweight/obesity found a lack of association, possibly due to wide age intervals examined (e.g. 41–78 y in one study [13] and 45–64 y in another [12]).

In Britain, there are limited data on population levels of sedentary behaviour, with no national data on time spent sitting at work or commuting. Moreover, comparison of associations for sedentary behaviour is hampered by differences in measurement of TV-viewing [11]–[13], [25] and total sitting time [18], [19]. Nonetheless, some consistencies are emerging in the literature. For example, our finding that, after adjustment for covariates, a unit increase in TV-viewing was associated with a mean increase in weight of 0.19 kg over the age interval 45–50 y (data not shown), is in line with a recent study of three cohorts showing an adverse influence of time spent watching TV on weight gain, with increases in time spent watching TV (per h/d) being associated with weight gain (approximately 0.14 kg) over a four-year period [32]. Our weaker evidence for a role of work sitting for BMI gain compared to the dose-response trend for TV-viewing is also consistent with the Nurses' Health Study, suggesting stronger effects for the latter: after adjusting for age, smoking and dietary habits, a 2 h/d higher time spent sitting at work was associated with a 5% higher obesity risk, compared to a 23% higher risk for TV-viewing [25].

However, while TV-viewing is a commonly used marker of sedentary behaviour, it is poorly understood. Associations between TV-viewing and BMI gain may reflect a true causal effect of sedentary behaviour, an association with other unhealthy behaviours or the influence of TV advertisements on unhealthy behaviours. Due to marked differences between TV-viewing and work sitting in associations observed with life-style and socioeconomic factors, we have previously suggested that TV-viewing may overestimate, and work sitting underestimate, the true association between sedentary behaviour and adiposity [20]. Given that most studies have focused on leisure-time recreation [33], which consists mainly of TV-viewing, current understanding of the role of sedentary lifestyles may be limited because of possible bias associated with this indicator. Alternatively, there may be differences in metabolic expenditure between the two sedentary behaviours, with a lower expenditure for TV-viewing than sitting doing office work [34]. Hence, stronger associations for TV-viewing may reflect the lower energy expenditure of this behaviour. The manner in which sedentary time is accumulated may be important [35], with a larger number of breaks in time spent being sedentary being associated with lower BMI, independently of overall sitting time. It is conceivable that more breaks are taken during work sitting than TV-viewing, with the consequence that, for similar levels of exposure, uninterrupted TV-viewing may be more detrimental to health.

The proportion commuting by motorised transport in our study (77%) is comparable to employed adults in southern Sweden commuting by car or public transport (75%) [36] and only slightly higher than those driving to work in New South Wales (69%) [37]. Similar to previous work [36], [37] we found cross-sectional associations between commuting method and BMI, with those using motorised transport having on average a higher BMI by 0.33 kg/m^2^. However, no prospective relationship was observed and to our knowledge, there is no population level prospective study examining commuting method and subsequent BMI. Furthermore our findings on commuting need to be interpreted with caution. Evidence suggests those commuting by public transport walk substantial distances to bus/railway stations [38] and we were unable to differentiate between those using public and private motorised transport.

### Implications and conclusions

In summary, we found an increasing trend of higher 45–50 y BMI gain with higher TV-viewing at 45 y and although no trend was seen for work sitting, there was a detrimental effect on BMI gain for those sitting for 2–3 h/d. We also found no association between commuting by motorised transport and subsequent BMI. Hence, our findings suggest that TV-viewing may be important with respect to BMI gain in mid-adulthood. In the USA the lowest and highest quintile of TV-viewing in 2011 was 1 h/d and 10 h/d respectively and the amount of time spent watching TV is increasing [5], [6]. Our findings imply that the level of watching TV, by large proportions of the population shown here and in previous studies [39]–[41], could lead to increased BMI gain. Given the increasing prevalence of sedentary behaviours and paucity of information on sedentary time outside of the leisure domain, further investigation is warranted to clarify the impact of sedentary behaviour from different domains on subsequent adiposity.

## References

[1] Thorp AA, Owen N, Neuhaus M, Dunstan DW (2011) Sedentary behaviors and subsequent health outcomes in adults a systematic review of longitudinal studies, 1996–2011. Am J Prev Med 41: 207–215. 21767729 10.1016/j.amepre.2011.05.004

[2] Department of Health PA, Health Improvement and Protection, (2011) Start Active, Stay Active: A report on physical activity from the four home countries' Chief Medical Officers.

[3] Canadian Society for Exercise Physiology (2012) Canadian Physical Activity Guidelines and Canadian Sedentary Behaviour Guidelines.

[4] Matthews CE, Chen KY, Freedson PS, Buchowski MS, Beech BM, et al (2008) Amount of time spent in sedentary behaviors in the United States, 2003–2004. Am J Epidemiol 167: 875–881. 18303006 10.1093/aje/kwm390 PMC3527832

[5] The Nielsen Company (2011) State of the Media TV Usage Trends: Q3 and Q4 2010. The Nielsen Company

[6] The Nielsen Company (2011) The Cross-Platform Report. The Nielsen Company

[7] Swinburn BA, Sacks G, Hall KD, McPherson K, Finegood DT, et al (2011) The global obesity pandemic: shaped by global drivers and local environments. Lancet 378: 804–814. 21872749 10.1016/S0140-6736(11)60813-1

[8] Ford ES, Li C, Zhao G, Tsai J (2011) Trends in obesity and abdominal obesity among adults in the United States from 1999–2008. Int J Obes (Lond) 35: 736–743. 20820173 10.1038/ijo.2010.186

[9] Craig R, Mindell J (2011) Health Survey for England 2010. Volume 1—Respiratory Health.

[10] Wang YC, McPherson K, Marsh T, Gortmaker SL, Brown M (2011) Health and economic burden of the projected obesity trends in the USA and the UK. Lancet 378: 815–825. 21872750 10.1016/S0140-6736(11)60814-3

[11] Blanck HM, McCullough ML, Patel AV, Gillespie C, Calle EE, et al (2007) Sedentary behavior, recreational physical activity, and 7-year weight gain among postmenopausal US women. Obesity 15: 1578–1588. 17557996 10.1038/oby.2007.187

[12] Meyer AM, Evenson KR, Couper DJ, Stevens J, Pereria MA, et al (2008) Television, physical activity, diet, and body weight status: the ARIC cohort. International Journal of Behavioral Nutrition and Physical Activity 5. 10.1186/1479-5868-5-68 PMC2647944 19091124

[13] Ching PLYH, Willett WC, Rimm EB, Colditz GA, Gortmaker SL, et al (1996) Activity level and risk of overweight in male health professionals. Am J Public Health 86: 25–30. 8561237 10.2105/ajph.86.1.25 PMC1380355

[14] Raynor DA, Phelan S, Hill JO, Wing RR (2006) Television viewing and long-term weight maintenance: Results from the national weight control registry. Obesity 14: 1816–1824. 17062812 10.1038/oby.2006.209

[15] Coakley EH, Rimm EB, Colditz G, Kawachi I, Willett W (1998) Predictors of weight change in men: Results from The Health Professionals Follow-Up Study. Int J Obes (Lond) 22: 89–96. 10.1038/sj.ijo.0800549 9504316

[16] Jeffery RW, French SA (1998) Epidemic obesity in the United States: are fast foods and television viewing contributing? Am J Public Health 88: 277–280. 9491022 10.2105/ajph.88.2.277 PMC1508201

[17] Ding D, Sugiyama T, Owen N (2012) Habitual active transport, TV viewing and weight gain: A four year follow-up study. Preventive Medicine 54: 201–204. 22342646 10.1016/j.ypmed.2012.01.021

[18] Ekelund U, Brage S, Besson H, Sharp S, Wareham NJ (2008) Time spent being sedentary and weight gain in healthy adults: reverse or bidirectional causality? Am J Clin Nutr 88: 612–617. 18779275 10.1093/ajcn/88.3.612

[19] van Uffelen JGZ, Watson MJ, Dobson AJ, Brown WJ (2010) Sitting Time Is Associated With Weight, but Not With Weight Gain in Mid-Aged Australian Women. Obesity 18: 1788–1794. 20111018 10.1038/oby.2009.511

[20] Pinto Pereira SM, Ki M, Power C (2012) Sedentary Behaviour and Biomarkers for Cardiovascular Disease and Diabetes in Mid-Life: The Role of Television-Viewing and Sitting at Work. PLoS One 7: e31132. 22347441 10.1371/journal.pone.0031132 PMC3276501

[21] Power C, Elliott J (2006) Cohort profile: 1958 British birth cohort (National Child Development Study). Int J Epidemiol 35: 34–41. 16155052 10.1093/ije/dyi183

[22] Atherton K, Fuller E, Shepherd P, Strachan DP, Power C (2008) Loss and representativeness in a biomedical survey at age 45 years: 1958 British birth cohort. J Epidemiol Community Health 62: 216–223. 18272736 10.1136/jech.2006.058966

[23] Wareham NJ, Jakes RW, Rennie KL, Mitchell J, Hennings S, et al (2002) Validity and repeatability of the EPIC-Norfolk Physical Activity Questionnaire. Int J Epidemiol 31: 168–174. 11914316 10.1093/ije/31.1.168

[24] Parsons TJ, Thomas C, Power C (2009) Estimated activity patterns in British 45 year olds: cross-sectional findings from the 1958 British birth cohort. Eur J Clin Nutr 63: 978–985. 19223916 10.1038/ejcn.2009.6

[25] Hu FB, Li TY, Colditz GA, Willett WC, Manson JE (2003) Television watching and other sedentary behaviors in relation to risk of obesity and type 2 diabetes mellitus in women. JAMA 289: 1785–1791. 12684356 10.1001/jama.289.14.1785

[26] Power C, Atherton K, Strachan DP, Shepherd P, Fuller E, et al (2007) Life-course influences on health in British adults: effects of socio-economic position in childhood and adulthood. Int J Epidemiol 36: 532–539. 17255345 10.1093/ije/dyl310

[27] Parsons TJ, Power C, Manor O (2001) Fetal and early life growth and body mass index from birth to early adulthood in 1958 British cohort: longitudinal study. BMJ 323: 1331–1335. 11739217 10.1136/bmj.323.7325.1331 PMC60670

[28] Saunders JB, Aasland OG, Babor TF, de la Fuente JR, Grant M (1993) Development of the Alcohol Use Disorders Identification Test (AUDIT): WHO Collaborative Project on Early Detection of Persons with Harmful Alcohol Consumption–II. Addiction 88: 791–804. 8329970 10.1111/j.1360-0443.1993.tb02093.x

[29] Sterne JA, White IR, Carlin JB, Spratt M, Royston P, et al (2009) Multiple imputation for missing data in epidemiological and clinical research: potential and pitfalls. BMJ 338: b2393. 19564179 10.1136/bmj.b2393 PMC2714692

[30] Bauman A, Ainsworth BE, Sallis JF, Hagstromer M, Craig CL, et al (2011) The Descriptive Epidemiology of Sitting A 20-Country Comparison Using the International Physical Activity Questionnaire (IPAQ). Am J Prev Med 41: 228–235. 21767731 10.1016/j.amepre.2011.05.003

[31] Nyholm M, Gullberg B, Merlo J, Lundqvist-Persson C, Rastam L, et al (2007) The validity of obesity based on self-reported weight and height: Implications for population studies. Obesity 15: 197–208. 17228048 10.1038/oby.2007.536

[32] Mozaffarian D, Hao T, Rimm EB, Willett WC, Hu FB (2011) Changes in diet and lifestyle and long-term weight gain in women and men. The New England journal of medicine 364: 2392–2404. 21696306 10.1056/NEJMoa1014296 PMC3151731

[33] Wareham NJ (2007) Epidemiological studies of physical activity and diabetes risk, and implications for diabetes prevention. Appl Physiol Nutr Metab 32: 778–782. 17622293 10.1139/H07-032

[34] Ainsworth BE, Haskell WL, Whitt MC, Irwin ML, Swartz AM, et al (2000) Compendium of physical activities: an update of activity codes and MET intensities. Med Sci Sports Exerc 32: S498–504. 10993420 10.1097/00005768-200009001-00009

[35] Healy GN, Dunstan DW, Salmon J, Cerin E, Shaw JE, et al (2008) Breaks in sedentary time - Beneficial associations with metabolic risk. Diabetes Care 31: 661–666. 18252901 10.2337/dc07-2046

[36] Lindstrom M (2008) Means of transportation to work and overweight and obesity: A population-based study in southern Sweden. Preventive Medicine 46: 22–28. 17706273 10.1016/j.ypmed.2007.07.012

[37] Wen LM, Orr N, Millett C, Rissel C (2006) Driving to work and overweight and obesity: findings from the 2003 New South Wales Health Survey, Australia. Int J Obes (Lond) 30: 782–786. 16404406 10.1038/sj.ijo.0803199

[38] Besser LM, Dannenberg AL (2005) Walking to public transit steps to help meet physical activity recommendations. Am J Prev Med 29: 273–280. 16242589 10.1016/j.amepre.2005.06.010

[39] Bertrais S, Preziosi P, Mennen L, Galan P, Hercberg S, et al (2004) Sociodemographic and geographic correlates of meeting current recommendations for physical activity in middle-aged French adults: the Supplementation en Vitamines et Mineraux Antioxydants (SUVIMAX) Study. American Journal of Public Health 94: 1560–1566. 15333315 10.2105/ajph.94.9.1560 PMC1448494

[40] Bertrais S, Beyeme-Ondoua JP, Czernichow S, Galan P, Hercberg S, et al (2005) Sedentary behaviors, physical activity, and metabolic syndrome in middle-aged French subjects. Obes Res 13: 936–944. 15919848 10.1038/oby.2005.108

[41] Stamatakis E, Hillsdon M, Mishra G, Hamer M, Marmot M (2009) Television viewing and other screen-based entertainment in relation to multiple socioeconomic status indicators and area deprivation: the Scottish Health Survey 2003. Journal of Epidemiology and Community Health 63: 734–740. 19401278 10.1136/jech.2008.085902

